# Non-Conventional Yeast Strains Increase the Aroma Complexity of Bread

**DOI:** 10.1371/journal.pone.0165126

**Published:** 2016-10-24

**Authors:** Elham Aslankoohi, Beatriz Herrera-Malaver, Mohammad Naser Rezaei, Jan Steensels, Christophe M. Courtin, Kevin J. Verstrepen

**Affiliations:** 1 Systems Biology Laboratory, VIB Center for Microbiology, Leuven, Belgium; 2 CMPG Laboratory of Genetics and Genomics, KU Leuven, Leuven, Belgium; 3 Laboratory of Food Chemistry and Biochemistry & Leuven Food Science and Nutrition Research Centre (LFoRCe), KU Leuven, Leuven, Belgium; University of Leicester, UNITED KINGDOM

## Abstract

*Saccharomyces cerevisiae* is routinely used yeast in food fermentations because it combines several key traits, including fermentation efficiency and production of desirable flavors. However, the dominance of *S*. *cerevisiae* in industrial fermentations limits the diversity in the aroma profiles of the end products. Hence, there is a growing interest in non-conventional yeast strains that can help generate the diversity and complexity desired in today’s diversified and consumer-driven markets. Here, we selected a set of non-conventional yeast strains to examine their potential for bread fermentation. Here, we tested ten non-conventional yeasts for bread fermentation, including two *Saccharomyces* species that are not currently used in bread making and 8 non-*Saccharomyces* strains. The results show that *Torulaspora delbrueckii and Saccharomyces bayanus* combine satisfactory dough fermentation with an interesting flavor profile. Sensory analysis and HS-SPME-GC-MS analysis confirmed that these strains produce aroma profiles that are very different from that produced by a commercial bakery strain. Moreover, bread produced with these yeasts was preferred by a majority of a trained sensory panel. These results demonstrate the potential of *T*. *delbrueckii* and *S*. *bayanus* as alternative yeasts for bread dough leavening, and provide a general experimental framework for the evaluation of more yeasts and bacteria.

## Introduction

Yeasts have been used for centuries for the production of fermented foods and beverages such as bread, wine and beer [1–3]. In ancient times, food fermentations were spontaneous processes. In the late 19^th^ century, however, spontaneous fermentations were gradually replaced by controlled processes where pure cultures were used as starter cultures, which yielded increased fermentation speed, quality and consistency. The predominant yeast used for such controlled fermentations is *Saccharomyces cerevisiae* because this species combines several desirable characteristics, including efficient and complete fermentation of high-sugar media, production of desirable flavors, absence of toxin production, and high ethanol production and tolerance [2, 4]. Currently, however, only a relatively limited number of genetically related and highly domesticated *Saccharomyces* strains are being used in industry, while much of the potential of the natural diversity of yeasts remains unexplored [2, 5]. For example, many of the currently used bakery strains are thought to have arisen from sexual crosses between a single ale and wine strain [6]. Hence, while use of a relatively homogenic group of *S*. *cerevisiae* yeasts for bread dough leavening has improved the speed, consistency and overall quality of fermentations, it also limited the sensorial complexity of the end product [7, 8]. One notable exception is the production of sourdough, where a rich microbial diversity (consisting of both yeasts and bacteria) coexists during the fermentation, resulting in unique sensorial features of the resulting bread [9].

The increasing interest in traditional and artisanal products, as well as the demand for niche products with distinctive aroma profiles is leading to a renewed interest into the potential of non-conventional microbes and spontaneous fermentations [2, 10, 11]. Many non-conventional yeasts produce unique aroma compounds that might be perceived as desirable in particular fermented products [12]. However, many non-conventional yeasts do not possess all the desirable qualities found in *S*. *cerevisiae*. Specifically, most yeasts fail to attain a desirable fermentation speed and attenuation. Alternative yeasts are therefore often not used as pure cultures, but rather mixed with *S*. *cerevisiae* or, alternatively used during pre-fermentation, before adding *S*. *cerevisiae* to complete the fermentation [2, 13–18].

Compared to the wine and beer industry, where the use of alternative yeasts has received considerable attention in the past years, the use of non-conventional strains for bread dough fermentation has received relatively little attention [19, 20]. This is likely due to the general, but mistaken, belief that bread yeast is only needed to provide the necessary carbon dioxide gas for leavening, while flavor compounds produced during fermentation would not contribute to the flavor profile of bread due to evaporation during baking. Recently, however, several studies have shown that yeast-derived compounds greatly contribute to the flavor profile of bread crumb [21, 22]. Moreover, beside the impact on flavor, some non-conventional strains show exciting characteristics for bread fermentation, such as freeze tolerance, amylase activity or the ability to ferment complex sugars [23, 24]. Even though many of these non-conventional yeasts are found in spontaneous fermentations, some can produce toxic compounds such as biogenic amines, products of amino acid decarboxylation that function as neurotoxins if absorbed in high concentrations [25]. Therefore, a careful test for production of any potentially harmful compounds by non-conventional yeasts is necessary before they can be employed in commercial food fermentations.

In this study, we selected 10 non-conventional yeast strains, investigated their performance in bread dough fermentation and determined their impact on the bread aroma profile, and measured their biogenic amine production. These 10 strains include two *Saccharomyces* species (*S*. *pastorianus* and *S*. *bayanus*) as well as 8 non-*Saccharomyces* strains (Table 1). We found that *Torulaspora delbrueckii and Saccharomyces bayanus* are very interesting for bread making because they combine acceptable dough fermentation capacity with the production of interesting aromas. Specifically, sensory analysis and HS-SPME-GC-MS analysis revealed a large difference in the aroma profile of bread fermented with these two strains compared to the control bread, fermented with a commercial bakery strain. Most importantly, bread fermented with these two non-conventional yeasts were preferred over conventional bread by a 20-person consumer panel sensory panel.

**Table 1:** Yeast species tested for their potential in bread making.

Strain number	Name	Source	Geographical origin
Y243	*Saccharomyces cerevisiae*	Baker’s yeast (control)	unknown
Y17	*Saccharomyces pastorianus*	Lager strain	Netherlands
Y156	*Saccharomyces bayanus*	Champagne	France
Y187	*Lachancea thermotolerans*	Wine	unknown
Y273	*Torulaspora delbrueckii*	Kaoliang mash	unknown
Y719	*Brettanomyces anomalus*	Cider	unknown
Y274	*Pichia kluyveri*	Cocoa	Java
Y276	*Wickerhamomyces subpelliculosa*	Cucumber brine	USA
Y281	*Pichia kudriavzevii*	Ginger beer	West Africa
Y494	*Pichia anomala*	Corn silage	Belgium
Y655	*Meyerozyma guilliermondii*	Wild	Belgium

## Materials and Methods

### Microbial culture

Because the aim of this study was to characterize non-conventional yeast strains that can be used as a main or auxiliary fermentation culture for bread making, we selected 10 strains with characteristics that are vital for dough fermentation, such as the ability to ferment maltose and complex sugars, and strains producing high concentrations of aroma-active compounds, such as isoamyl acetate and phenolic compounds. A genetically diverse set of yeast strains, consisting of species that are linked to food and/or beverage fermentations, was selected for these experiments. These strains include two *Saccharomyces* (non-*cerevisiae*) strains that are currently not used in bakery applications and eight non-*Saccharomyces* strains (Table 1). In addition, we used a commercial *Saccharomyces cerevisiae* bakery strain (Y243) as a control. The identity of the strains was confirmed using sequence analysis of the ITS region and D1/D2 region of the 26S rDNA gene. Yeast cultures were grown in YPD medium (20 g/l Yeast extract, 40 g/l bacterial Peptone, 20 g/l Dextrose at 30°C), using standard procedures as described previously [26, 27], and were harvested at early stationary phase and washed with water before inoculation into dough. The cell density was measured using a spectrophotometer based on the Optical Density measured at 600 nm.

### Population growth measurements

Yeast cells were harvested at the early stationary phase. In order to estimate the time needed for different strains to grow to the stationary phase, we followed the growth of all strains as a function of time. Cells from a turbid culture grown in YPD were inoculated in 150 microliter of YPD (to a final density of 1×10^5^ cells per ml) and allowed to grow in the Bioscreen C (Growth curves USA) at 30°C with continuous shaking until the stationary phase. The automated OD meter was set to read the OD_600_ every 15 min.

### Biogenic amine production

To confirm that the non-conventional yeast strains shortlisted here (Table 1) are safe to use, we checked that none produced any biogenic amines (BAs). Production of BAs was determined using an adapted version of the method explained by Joosten and Northholt [28, 29]. Briefly, yeast strains (10^6^ cells per ml) were inoculated onto YPD agar plates supplemented with bromocresol purple (Sigma Aldrich) 0.006% and an amino acid mix with a total mass concentration of 1% (MP Biochemicals, LLC). The added amino acids are tyrosine, histidine, phenylalanine, leucine, tryptophan, arginine and lysine at equal ratios. Subsequently, the plates were incubated at 30°C for 7 days and the growth and changes in the color of the medium was monitored daily to test for the presence of BAs. In strains with no BA production, the growth area was surrounded by a yellow halo caused by glucose fermentation, followed by a pH reduction that causes the medium to turn purple after a period that depends on the growth rate of the strain. By contrast, when BAs are produced, amino acid decarboxylation resulted in a purple halo from the very beginning, which grew bigger and darker as a function of time.

### Dough preparation and fermentation monitoring

Commercial wheat flour (Ceres-Soufflet, Brussels, Belgium) was used for this part of the study. Dough was prepared according to the straight-dough method using the following formula: per 100.0 g flour (on a 14% moisture basis), 6.0% (w/w) sucrose, 1.5% (w/w) sodium chloride, 52.0% (v/w) water, and 5.3% (w/w) fresh yeast pellet (16.0 ± 0.5% dry matter) [30]. All doughs were prepared in duplicate.

All ingredients were mixed in a 10-g pin bowl mixer (National Manufacturing, Lincoln, NE, USA) for 3 min 50 s. Next, the volume of CO_2_ gas produced by the different strains during dough fermentation was measured using a Risograph instrument (National Manufacturing). Balls of dough, made as described above, were allowed to ferment in the instrument for 5 hours at 30°C. Gas production was measured continuously at 1-min intervals.

### Production of Bread

For the sensory and GC-MS analysis, we prepared loaves of bread with the following formula using an automatic bread maker (Panasonic SD-ZB2502): 400 g flour (11.5/680, commercial wheat flour by Soubry, Roeselare, Belgium), 4% (w/w) yeast, 3% (w/w) sugar, 1.5% (w/w) salt and 230 ml water. This formula was used both for the control as well as all other strains that were able to ferment dough with an acceptable efficiency. To obtain a completely fermented dough with the 2 strains that were unable to satisfactorily ferment dough (*Brettanomyces anomalus* (Y719) and *Saccharomyces pastorianus* (Y17)), 2% (w/w) bakery strain (control strain) was supplemented with 2% (w/w) of Y719 or Y17. In this case, we determined the effect of adding these strains to the conventional starter culture with 4% baker’s yeast.

The Panasonic SD-ZB2502 apparatus was set at program 01 (Basic), which is 4 hours long including the 2 hours 50 min fermentation time, i.e. the time between adding the yeast and the start of the baking. After the completion of program, the bread was removed from the machine and allowed to cool to the room temperature for 3 hours. Next, a bread slicer (BOSCH MAS4201) was used to make equal slices (15 mm thick) of bread for sensory analysis. For each biological replicate (bread made on a different day, n = 2), bread crumb samples were taken from the center of four slices drawn from different random sections of the bread to have representative bread samples. The GC-MS samples (5.00 gr bread crumb) were immediately stored in sealed GC-MS vials at -20°C for later analysis. The size of bread loaves was compared based on their height since the baking tray keeps the other dimensions equal between all loaves.

### Sensory analysis

The bread made with the different yeast strains was subjected to sensory analysis using the triangle test [31]. The aim of this test was to determine whether the aroma as well as the taste of any given sample of bread prepared using a non-conventional yeast strain could be discriminated by a consumer test panel from that produced by control samples (bread made with commercial bakery strain Y243). To evaluate bread made with each of the different yeasts, we employed a classic triangle test with a panel of twenty participants for both the aroma as well as the taste tests.

For the triangle tests, panelists were instructed to evaluate three samples, of which two were identical and one was different. The serving order was randomized for each panel member, but care was taken to present all possible serving orders (AAB, ABA, BAA, BBA, BAB, ABB). Each sample consisted of a 4 x 4 cm 15 mm thick slice of bread, which did not contain any crust. Samples were served in small plates at room temperature (23°C) and were coded with a three-digit random number. The blindfolded panelists were asked to identify which one of the samples was different from the others, based first only on their aroma (smell) and second based on their taste. The panelists were also asked if they had a preference for one of samples based on the overall flavor, and explained their decision using descriptors. If a panelist was unable to detect any difference between any three samples, they were not asked about their preference. Water was served for neutralization in between samples.

Significance was assessed using binomial Generalized Linear Model (GLM) by testing whether the proportion of correctly recognized samples differed significantly from the 1/3 probability expected by chance alone. Using the results of this first triangle test, we shortlisted the most promising strains and carried out an additional triangle test for further confirmation. Overall significance in this case was assessed using a binomial GLM, in which replicate was included as a fixed factor, and we again tested for an overall deviation from 1/3. These analyses were performed using function glm in R version 3.01. GC-MS was used to investigate differentially produced volatile compounds for the samples that showed significant differences with the control based on the result of the triangle test.

### Analysis of bread volatile compounds

Sensory analysis demonstrated that the aroma of bread fermented with *S*. *bayanus* and *T*. *delbrueckii* could be discriminated from the bread baked using the commercial bakery strain Y243. Hence, the volatiles of the bread fermented using these two strains were compared with those of the bread prepared using the commercial bakery strain (control). The bread aroma profile was analyzed using headspace solid-phase micro extraction (HS-SPME) followed by gas chromatography–mass spectrometry (GC–MS). For each SPME sample, 5.00 g of bread crumb was put in a 20 ml glass vial which was sealed with a silicone septum and kept in -20°C before analysis. After leaving the vial for 30 minutes at room temperature to thaw, the vial was immersed in a water bath at 40°C. After 5 min of equilibration, a triphase DVB/Carboxen/PDMS 50/30 μm SPME fiber (Supelco Co., Bellefonte, PA, USA) was used to sample volatiles for 30 min (cf. [32]). Subsequently, compounds trapped on the fiber were thermally desorbed in the injection port of a gas chromatograph-mass spectrometer (Shimadzu QP2010 Ultra Plus) by heating the fiber for 5 min at 250°C. For each strain, two biological replicates were analyzed, using four technical replicates each sampled across different slices of the bread. For each biological replicate of *S*. *bayanus* and *T*. *delbrueckii*, we also prepared a matching control, i.e. samples of bread prepared with the baker’s yeast control strain on the same day.

The GC-MS was equipped with a HP-5ms non-polar column (Agilent, 30 m × m x 0.25 mm i.d, 0.25 μm thin layer). Helium was used as carrier gas with a flow rate of 1.4 ml/min. Manual injection was carried out in splitless mode. The temperature was first held at 36°C for 10 min and then allowed to rise to 220°C at a rate of 6°C/min. The mass detector was operated in scan mode (35–600 amu), using electron impact ionization (70 eV). The interface and detector temperatures were kept at 250°C. A mix of linear *n*-alkanes (from C_8_ to C_19_) were injected into the GC-MS under identical conditions to serve as external retention index markers. Subsequently, accurate retention indices of all volatile compounds were calculated using cubic spline interpolation [33].

Compound spectra were deconvoluted using AMDIS version 2.71 and matched to commercial GC/MS libraries such as FFNSC version 1.3, the Adams 4^th^ edition essential oil library and NIST/EPA/NIH Mass Spectral library version 2011 [34–36]. Retention indices (RI) of pure standards and of compounds reported in the literature, in the NIST 2011 retention index database and Flavornet were used as additional criteria to confirm the identity of each compound. Since some compounds coeluted with one another, analyses was performed by integrating over characteristic ions (Table 2) using OpenChrom (version 0.9.0). Subsequently, integrated areas were converted back to a total ion current scale based on the relative abundance of each characteristic ion in the standard spectrum of each respective compound. Relative quantification was determined based on the peak areas for each volatile relative to the sum of all metabolites abundances in the sample. To test for differences in the volatiles released by bread made using the non-conventional yeast strains *T*. *delbrueckii* (Y273) and *S*. *bayanus* (Y156), and their respective baker’s yeast controls we statistically compared the log transformed relative peak areas (the log of the peak area divided by the total peak areas of all compounds present in each sample) using linear mixed models in which biological replicate was coded as a random factor and strain and day were coded as fixed factors. Subsequently, the significance of differences in the relative quantity of each compound produced by bread prepared with the different strains were obtained using Tukey posthoc tests. These analyses were performed using packages nlme and glmulti within R version 3.2.1.

**Table 2:** Volatile compounds identified and quantified in bread crumb of breads made with two non-conventional strains (*Torulaspora delbrueckii* Y273 and *S*. *bayanus* Y156) or commercial baker’s yeast (control Y243) using HS-SPME-GC-MS.

Chemical group	Compound	RI *	Ion (m/z) **	Odor ***	Y273	*Y156*	Y243
**Alcohols**	2-methyl-1-propanol	615	74	glue, alcohol	x	x	x
	3-methyl 3-buten-1-ol	696	*nq*	fruit, green	x	x	-
	3-methyl-1-butanol	704	57	whiskey, malt, alcohol	x	x	x
	2-methyl-1-butanol	708	56	malt, alcohol, balsamic	x	x	x
	1-pentanol	746	42	fruit	x	x	x
	2,3-butanediol	794	45	butter, cream	x	x	x
	3-ethoxy-1-propanol	850	59	fruit	x	x	x
	1-hexanol	855	69	flower, green-grass	x	x	x
	1-heptanol	971	70	mushroom, green	x	x	x
	1-octen-3-ol ^a^	980	100	mushroom	x	x	x
	2-phenylethanol	1111	122	honey, rose, flower	x	x	x
	3Z-nonen-1-ol	1154	95	NA	x	x	x
	4Z-decen-1-ol	1259	67	NA	x	x	x
	dihydromyrcenol	1072	123	tart lime, citrus	-	x	x
**Aldehydes**	3-methyl butanal	633	44	malt, fermented, cocoa	x	x	x
	2-methyl butanal	636	41	malt	x	x	x
	Hexanal	792	44	grass, green	x	x	x
	heptanal	899	70	fat, rancid, pungent	x	x	x
	benzaldehyde ^a^	955	105	almond	x	x	x
	Octanal	1002	84	lemon, citrus	x	x	x
	phenylacetaldehyde ^a^	1042	120	honey, sweet	x	x	x
	nonanal	1104	69	fruit, soap, citrus	x	x	x
	2E-nonen-1-al ^a^	1160	83	fat, cucumber, green	x	x	x
	safranal	1199	107	herb, sweet	x	x	x
	decanal	1207	55	soap, orange peel	x	x	x
**Esters**	3-methyl-1-butanol acetate	881	70	banana	x	x	x
	ethyl octanoate	1196	88	fruit, fat, sweet, soap	x	x	x
	2-phenylethyl acetate	1256	104	rose, honey, flower	x	x	x
	ethyl decanoate	1394	60	grape, fruit	x	x	x
	ethyl dodecanoate	1593	88	leaf	x	x	x
**Ketones**	2,3-butanedione	595	86	butter	x	x	x
	2,3-pentadione	660	*nq*	cream, butter	x	x	x
	3-hydroxy-2-butanone	678	45	cream, butter	x	x	x
	2-heptanone	890	58	soap, fruit, cinnamon	x	x	x
	1-octen-3-one	976	70	mushroom	x	x	x
	6-methyl-5-hepten-2-one	983	108	green, citrus	x	x	x
	2-octanone	989	58	herb, unripped apple	x	-	-
	acetophenone	1066	105	must, flower, almond	x	x	x
	2-nonanone	1091	57	fruit, flower	x	x	x
	isophorone	1120	138	peppermint-like	x	x	x
**Acids**	acetic acid ^a^	618	60	sour, pungent, vinegar	x	x	x
	3-methyl-butanoic acid	872	*nq*	cheese, sweat, rancid	x	x	x
	2-methyl-butanoic acid	876	74	cheese, sweat	x	-	x
	2-ethyl-hexanoic acid	1118	73	mild	x	x	x
	octanoic acid	1174	60	sweat, soap, fruit-acid	x	x	x
	nonanoic acid	1270	60	green, fat	x	x	x
**Terpenes**	limonene ^a^	1026	68	citrus, lemon, mint	x	x	x
	geranyl acetone ^a^	1448	43	magnolia, green	x	x	x
	longifolene	1416	161	NA	x	x	x
	caryophyllene-E	1425	93	wood, spice	x	x	x
**Furans**	2-ethyl furan	661	96	malt, sweet	x	x	x
	furfural	838	*nq*	bread, almond, sweet	x	x	x
	2-furanmethanol	864	98	burnt, warm oil	x	x	x
	2-pentyl furan	987	138	fruit, flower	x	x	x
**Lactones**	gamma-hexalactone	1053	85	caramel, nut, malt	x	x	x
	gamma-nonalactone	1363	85	coconut, sweet, cream	x	x	x
**Alkanes & alkenes**	Hexane ^a^	600	56	gasoline	x	x	x
	Dodecane ^a^	1200	85	NA	x	x	x
	Tetradecane ^a^	1400	57	NA	x	x	x
**Others**	dimethyl trisulfide	961	126	cabbage, sulfury	x	x	x
	para-cymene ^a^	1021	119	solvent, citrus	x	x	x
	para-vinyl-guaiacol^a^	1317	135	clove, spices	x	x	x

Compounds marked with an “x” were detected in the bread crumb produced with the corresponding strain, while “-”marks their absence.

* Retention Index (RI) measured by GC-MS with non-polar column, and calculated using cubic spline interpolation (Halang et al. 1978).

^a^ Compounds for which RIs and mass spectra were confirmed with pure standards.

** Mass fragment used for quantification. *nq*: compound not quantified due to the poor reproducibility of their area, low signal to noise rate or the absence of unique diagnostic ions. NA: not available information.

*** Odor descriptions were taken from [38–44].

### Nucleotide sequence accession numbers

The DNA sequences of representative isolates of the different species have been deposited in GenBank (accession numbers KX021887 to KX021902).

## Results

### Evaluation of non-conventional yeast strains for bread dough fermentation

**Fig 1:**
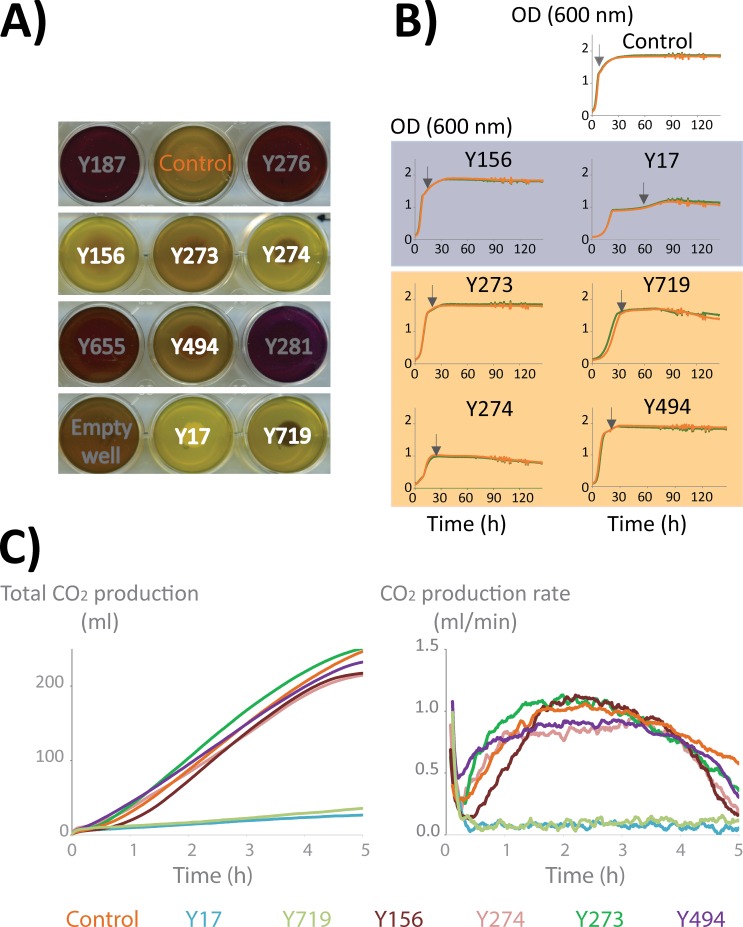
Small-scale fermentation tests for ten selected nonconventional yeasts. (A) Four strains (shown in gray font) were found to produce biogenic amines. (B) Small-scale growth assays using a Bioscreen C apparatus shows the growth curves of the different strains, the arrows show the harvest point (late diauxic shift/ early stationary phase). (C) CO_2_ production of the six strains that did not show biogenic amine production during dough fermentation as measured in the Risograph.

To select suitable non-conventional yeast strains for bread making, we first tested whether the 10 shortlisted strains (Table 1) were safe to use according to literature and legislation: did not produce any biogenic amines (BAs) and had satisfactory fermentation characteristics. Biogenic Amine (BA) production was tested using a modified version of the Joosten and Northholt method [28, 29]. This test resulted in the elimination of *Lachancea thermotolerans* (Y187), *Wickerhamomyces subpelliculosa* (Y276), *Wickerhamomyces subpelliculosa* (Y655) and *Pichia kudriavzevii* (Y281), as they were found to produce (small amounts of) biogenic amines (Fig 1A).

**Fig 2:**
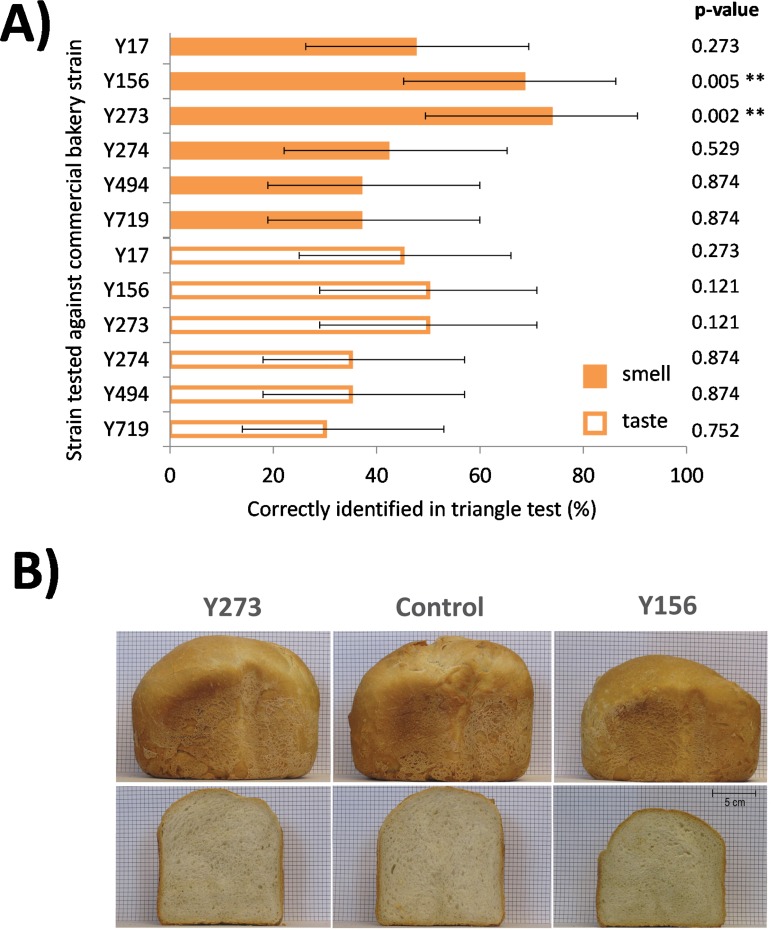
Sensory analysis and leavening ability of non-conventional yeasts compared to commercial bakery control yeast. (A) Triangle tests with a 20-person consumer panel show that out of six bread samples produced with different yeasts, two samples (fermented with *Torulaspora delbrueckii* Y273 and *S*. *bayanus* Y156) yielded bread that was recognized as being significantly (** = *p* < 0.01) different by the sensory panel members when compared to bread produced with the control commercial bakery strain. The difference in smell was more pronounced than the difference in taste. Bar graphs represent the percentage (± 95% confidence limits) of participants that correctly identified the odd sample in a triangle test and noticed the difference between the sample and the control. Significance levels were calculated using binomial tests based on the deviation from the 33% of correct identifications as expected by chance. (B) Fermentation with Y156 results in a slightly smaller loaf of bread compared to the control while the volume of the bread fermented by Y273 is comparable to that of the control.

In order to obtain optimal yeast performance for dough fermentation, it is important to harvest the cells in late diauxic shift or early stationary phase [37]. We followed the growth curve of the six remaining strains to find the best OD for cell harvest before inoculation in dough (Fig 1B). Next, we tested the bread dough fermentation capacity of the six remaining strains by measuring CO_2_ production as a proxy for fermentation capacity using a Risograph. These tests demonstrated that *S*. *bayanus* (Y156), *T*. *delbrueckii* (Y273), *B*. *anomalus* (Y719) and *P*. *anomala* (Y494) ferment dough with a fermentation capacity similar to that of the commercial control strain (Fig 1C), whereas two other strains tested (*Saccharomyces pastorianus* (Y17) and *Brettanomyces anomalus* (Y719)) were unable to produce CO_2_ and leaven dough (Fig 1C). Specifically, fermentation of dough with *S*. *bayanus* (Y156) resulted in a slightly smaller loaf of bread compared to bread produced with the reference baker’s yeast, while the size of bread fermented with *T*. *delbrueckii* (Y273) was comparable to that of control bread (Fig 2B). In addition, out of the four most promising remaining strains, two (*S*. *bayanus* (Y156) and *T*. *delbrueckii* (Y273)) yielded excellent aroma profiles (see below and Fig 2A).

### Sensory analysis

As a next step in evaluating the suitability of using non-conventional yeasts for bread dough fermentation, a 20-person consumer panel helped to assess if and how the breads fermented with the non-conventional strains were perceived as being different from the bread made with the control bakery strain in terms of their aroma and taste, and if the non-conventional yeasts yielded bread that was preferred over that made with conventional baker’s yeast. The four strains that show satisfactory dough fermentation capacities were inoculated in dough as pure culture, while the two strains that did not efficiently ferment dough were used in a mixed culture (50%-50%) together with a commercial bakery strain (Y243). Subsequently, the aroma and taste of the bread made using the non-conventional yeast strains were compared to the control samples, bread fermented with the pure commercial bakery strain, using triangle tests.

The results of these sensorial tests demonstrate that two out of the six strains (*S*. *bayanus* (Y156) and *T*. *delbrueckii* (Y273), both used as pure culture) yielded bread that was perceived as having a significantly different aroma compared to the control produced with a commercial bread yeast strain (binomial GLM; p < 0.01) (Fig 2A). The validation triangle tests performed using these two strains confirmed these results, and demonstrated that bread made with the Y156 or Y273 strain was perceived as being significantly different from the control (p = 0.016 and p = 0.005, respectively, based on binomial GLMs). In contrast to these clear perceived differences in odor, the taste was not perceived as significantly different compared with the control by our panel for either of the strains (Y156: p = 0.121 and Y273: p = 0.273, binomial GLM). For the *T*. *delbrueckii* strain Y273, 57.5% of the participants noticed the difference with the control across both replicate trials, and out of these, eight had an overall preference for the taste and aroma of the bread prepared using strain Y273, ten had no preference and five preferred the control. The participants described the control as having a regular bread smell whereas the bread prepared using the *T*. *delbrueckii* strain Y273 was described as having a more complex, nutty, forest-like flavor reminiscent of some breads prepared by spontaneous fermentation processes. For the *S*. *bayanus* strain Y156, 60% of the panelists noticed the difference with the control across both replicate trials and six of them preferred the bread prepared using strain Y156, 13 had no strong preference and five preferred the bread produced with the control yeast. In this case, the control was described as having a regular bread flavor while the bread prepared using the *S*. *bayanus* strain Y156 was described as slightly more aromatic and fruity.

### Characterization of differentially produced aroma compounds

GC-MS analysis of bread crumb samples identified 62 different volatile compounds (Table 2). Out of these, 59 were sufficiently abundant (signal to noise ratio higher than 10) to enable comparing their relative abundances in bread prepared using *S*. *bayanus* or *T*. *delbrueckii* and that prepared using the control bakery strain (Table 2).

**Fig 3:**
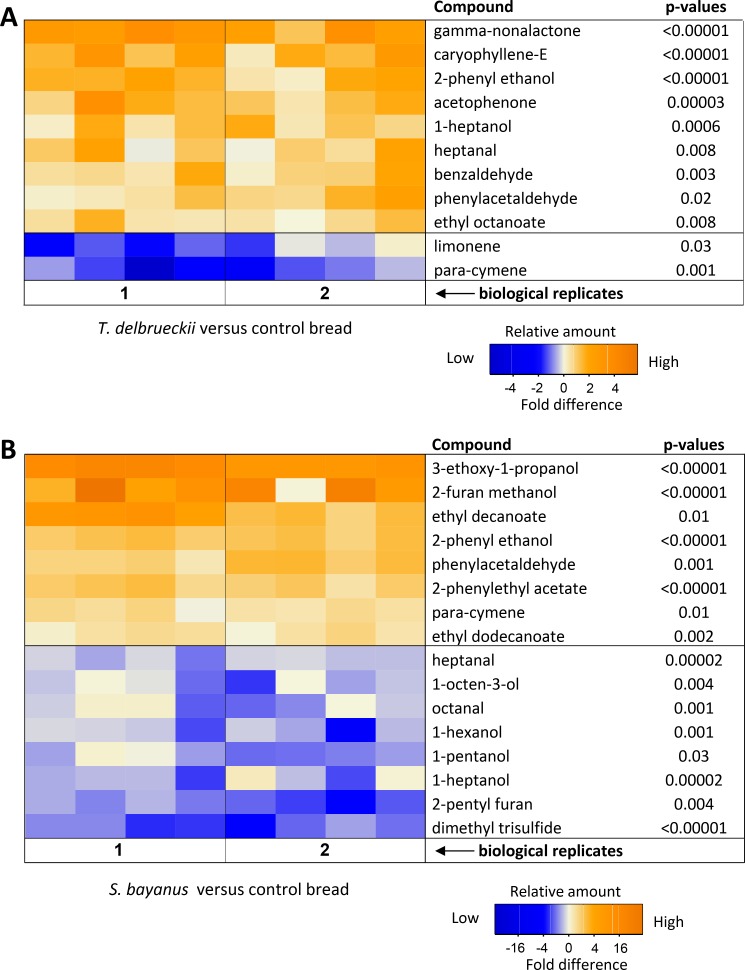
**Heatmaps illustrating the differences in relative concentrations of volatile compounds in bread crumb prepared with nonconventional yeasts (A) *Torulaspora delbrueckii* (Y273) and (B) *S*. *bayanus* (Y156) compared to bread produced with a commercial baker strain (control).** Data are based on HS-SPME-CG-MS analysis of two biological replicates with four technical replicates each. Color codes indicate the fold difference in log2-transformed relative peak areas of each compound, between samples and their controls. *P*-values were obtained using a linear mixed models and Tukey posthoc analysis, in which biological replicate and treatment were coded as random and fixed factors.

In bread prepared using *T*. *delbrueckii* (Y273), the relative concentration of nine compounds (gamma-nonalactone, caryophyllene-E, 2-phenyl ethanol, acetophenone, 1-heptanol, heptanal, benzaldehyde, phenylacetaldehyde and ethyl octanoate) was significantly elevated compared to the control (Fig 3, Tukey HSD test, p < 0.05). Out of these, 2-phenyl ethanol, 1-heptanol, heptanal, benzaldehyde, phenylacetaldehyde and ethyl octanoate have previously been reported to be important aroma compounds in bread crumb [21, 22, 45–47]. By contrast, only two compounds were found to be present in significantly higher concentrations in the control, namely limonene and para-cymene (Fig 3A).

From the compounds observed in higher amounts in bread produced using *T*. *delbrueckii*, 1-heptanol and heptanal are derived from the oxidation of lipids and have previously been identified as being important for bread crumb flavor (Table 3). Moreover, benzaldehyde and 2-pentyl furan have been shown to result from lipid degradation and the fermentative activity of the yeast (Table 3) [45]. In addition, 2-pentyl furan has also been reported to be produced in Maillard reactions. This compound has been reported as the most important furan in bread crumb [22].

**Table 3:** Overview of compounds that showed differences between breads produced with two nonconventional strains (*Torulaspora delbrueckii* Y273 and *S*. *bayanus* Y156) and commercial baker’s yeast (control Y243). All of these compounds have Odor Activity Values** (OAV) > 0.1 and Flavor Dilution*** (FD) factors > 8 [45].

Chemical group	Compound	Origin	Odor threshold * (OT) in water (μg/kg)
**Alcohols**	1-heptanol	Lipid degradation	3
	1-octen-3-ol	Lipid degradation	1
	2-phenylethanol	Fermentation	1100
**Aldehydes**	heptanal	Lipid degradation	3
	phenylacetaldehyde	Fermentation and Maillard reaction	4
	benzaldehyde	Fermentation and Lipid degradation	350
	octanal	Lipid degradation	0.7
**Ester**	ethyl octanoate	Fermentation	92
**Furan**	2-pentyl furan	Fermentation, Lipid degradation and Maillard reaction	6

* Odor threshold (OT) in water compiled from [45].

** Odor Activity Value (OAV) is the ratio of the compound concentration and its OT in water

*** Dilution Factor (FD) factor is the ratio between the concentration of each compound in the initial extract to its concentration in the most dilute extract in which its odor can be detected by GC-Olfactometry.

Bread made with *T*. *delbrueckii* contained more carbonyl compounds compared to breads produced by the control commercial bakery strain. High levels of aldehydes and ketones are typically associated with unfermented raw materials or samples at earlier stages of fermentation [48–50], suggesting that fermentation with *T*. *delbruekii* resulted in a slower reduction of these carbonyl compounds to corresponding alcohols, esters and acids.

Comparison of the concentration of volatile compounds in breads made with *S*. *bayanus* versus the control revealed significant differences in 16 compounds (Fig 3B, Tukey HSD test, p < 0.05). Half of the compounds show higher concentrations in the samples made with *S*. *bayanus* compared to the commercial bakery strain (Fig 3B, Tukey HSD test, p < 0.05). Out of the compounds that show significant differences in bread prepared with *S*. *bayanus* and the control, six are known as key aroma compounds in bread crumb [45]. The list also includes phenylacetaldehyde and 2-phenylethanol, which are known to have very low odor threshold (OT) values (Table 3). These compounds are formed inside the yeast cell through degradation of flour amino acids via the Ehrlich pathway [50], specifically from degradation of phenylalanine.

Volatile esters are a particularly important class of compounds because these yeast-derived molecules are responsible for highly desired fruity aroma in fermentation products [51–53] (Table 2). In particular, we found that ethyl octanoate, 2-phenylethyl acetate, ethyl decanoate and ethyl dodecanoate are present in significantly higher concentrations in the bread crumb prepared using the non-conventional *S*. *bayanus* and *T*. *delbruekii* yeasts compared to the bread prepared using the control bakery yeast (Fig 3). Furthermore, 2-phenylethyl acetate has been described as one of the major esters produced during dough fermentation [54].

Importantly, although it has been shown that there is variability in bread aroma produced by different *S*. *cerevisiae* strains [21, 55], many compounds for which we observed quantitative differences between the non-conventional strains and the commercial baker yeast control did not show any differences in breads prepared with different *S*. *cerevisiae* strains. For example, increased production of 1-heptanol and heptanal was clearly associated with the use of *T*. *delbruekii*, whereas reduced production of 1-hexanol, 1-heptanol, heptanal, octanal and 2-pentyl furan were associated with bread made with *S*. *bayanus*, and none of these compounds were found to vary across bread prepared with different *S*. *cerevisiae* strains [21], suggesting that, compared to standard baker’s yeast, nonconventional strains cannot only produce different concentrations of aroma compounds, but also an entire set of aroma compounds that are not produced by the standard yeast.

## Discussion

We tested several non-conventional yeast strains for their potential as agents for bread dough fermentation, with the specific aim to generate bread with a distinct, desirable flavor. We identified two strains (from the species *T*. *delbrueckii* and *S*. *bayanus*) that show great potential and produce breads that show different aroma profiles from those produced with a standard commercial bakery strain. Importantly, a sensory panel was also able to recognize the differences between breads produced by standard and nonconventional yeasts. Moreover, the majority of our consumer test panel preferred breads made with these special yeasts over the standard bread. Furthermore, the sensorial descriptions given by the panel members are consistent with the differences that we measured by GC-MS in the volatile compounds in the bread crumb samples.

A wide range of volatile compounds was identified and quantified in the dough samples (Table 2), including several alcohols, aldehydes, ketones, esters, acids, terpenes, furan derivatives, pyrazines, alkanes, phenols and a sulfur compound. We identified more pronounced differences in the concentrations of flavor compounds in bread fermented with *S*. *bayanus* than in bread fermented with *T*. *delbrueckii* compared to the control breads (Fig 3). Both non-conventional yeasts have already been suggested as being potentially interesting for bread making. Specifically, *S*. *bayanus* has been suggested to reduce over-leavening of dough [56]. *T*. *delbrueckii* has been shown to be osmo- and cryotolerant [23, 24] with better leavening ability of sweet and frozen dough compared to *S*. *cerevisiae* [57]. However, despite these previous reports, the two yeasts are not commonly used in industrial bread making. Their positive effects on bread flavor and their potential to make flavorful standard breads were not recognized. While the standard baker’s yeast *S*. *cerevisiae* is one of the best-studied model organisms, most of the other yeasts are understudied and, as a result, underappreciated; except perhaps for a few notable exceptions [2].

Our results are in line with other recent studies that report the importance of yeast and the fermentation process for the final aroma profile of bread [21, 22]. Even though the majority of the aroma compound in bread crust is the result of baking in the oven, and in particular the Maillard reactions that take place during this heating step [58], the crumb’s sensorial characteristics are also shaped by the fermentation and the choice of yeast. Our data are also consistent with a previous study that shows that both *T*. *delbrueckii* and *S*. *bayanus* produce different aroma profile compared to standard bakery strains in liquid fermentation [59]. In particular, the aroma of preferment and bread using *T*. *delbruekii* were described as very special compared to those of baker’s yeast [59].

## Conclusion

Together, our results demonstrate that *T*. *delbrueckii* and *S*. *bayanus* are interesting candidates for application in the baking industry. Our data show that these two strains add more complexity to the sensory profile of bread, adding specific nutty and fruity tones. More generally, although we screened only a limited number of non-conventional strains, our data suggests that there is an exciting potential for non-conventional strains to be used in the baking industry in order to increase the diversity of the product and match the requirements of particular customers. Moreover, the experiments and screening methods used in this paper could serve as an example to start large-scale studies aimed at identifying interesting non-conventional microbes for bread dough fermentation. As such, these results may propel further research in the use of alternative, non-conventional microbes for bread fermentation, similar to the recent efforts to find alternative microbes for beer and wine fermentation [60, 61].

## Supporting Information

S1 Fig CO_2_ production of nonconventional yeasts *Torulaspora delbrueckii* (Y273) and *S*. *bayanus* (Y156) compared with a commercial bakery strain (control) as measured in the Risograph (replicates of each strain are identify as A and B).
(TIF)

## References

[1] Sicard D, Legras JL. Bread, beer and wine: yeast domestication in the *Saccharomyces* sensu stricto complex. Comptes rendus biologies. 2011;334(3):229–36. Epub 2011/03/08. 10.1016/j.crvi.2010.12.016 . 21377618

[2] Steensels J, Verstrepen KJ. Taming wild yeast: potential of conventional and nonconventional yeasts in industrial fermentations. Annual review of microbiology. 2014;68:61–80. Epub 2014/04/30. 10.1146/annurev-micro-091213-113025 . 24773331

[3] Legras J-L, Merdinoglu D, Cornuet J-M, Karst F. Bread, beer and wine: *Saccharomyces cerevisiae* diversity reflects human history. Molecular Ecology. 2007;16(10):2091–102. 10.1111/j.1365-294X.2007.03266.x . 17498234

[4] Piskur J, Rozpedowska E, Polakova S, Merico A, Compagno C. How did *Saccharomyces* evolve to become a good brewer? Trends in Genetics. 2006;22(4):183–6. 10.1016/j.tig.2006.02.002 . 16499989

[5] Gallone B, Steensels J, Prahl T, Soriaga L, Saels V, Herrera-Malaver B, et al Domestication and divergence of *Saccharomyces cerevisiae* beer yeasts. Cell. 2016;In Press. 10.1016/j.cell.2016.08.020 PMC5018251 27610566

[6] Randez-Gil F, Corcoles-Saez I, Prieto JA. Genetic and Phenotypic Characteristics of Baker's Yeast: Relevance to Baking. Annu Rev Food Sci Technol. 2013;4:191–214. 10.1146/annurev-food-030212-182609 . 23464571

[7] Domizio P, Lencioni L, Ciani M, Di Blasi S, Pontremolesi C, Sabatelli MP. Spontaneous and inoculated yeast populations dynamics and their effect on organoleptic characters of Vinsanto wine under different process conditions. International Journal of Food Microbiology. 2007;115(3):281–9. 10.1016/j.ijfoodmicro.2006.10.052 . 17307268

[8] Daenen L, Sterckx F, Delvaux FR, Verachtert H, Derdelinckx G. Evaluation of the glycoside hydrolase activity of a *Brettanomyces* strain on glycosides from sour cherry (*Prunus cerasus* L.) used in the production of special fruit beers. Fems Yeast Research. 2008;8(7):1103–14. 10.1111/j.1567-1364.2008.00421.x . 18673394

[9] De Vuyst L, Van Kerrebroeck S, Harth H, Huys G, Daniel HM, Weckx S. Microbial ecology of sourdough fermentations: diverse or uniform? Food Microbiology. 2014;37:11–29. Epub 2013/11/16. 10.1016/j.fm.2013.06.002 . 24230469

[10] Rainieri S, Pretorius IS. Selection and improvement of wine yeasts. Annals of Microbiology. 2000;50(1):15–31. .

[11] Schuller D, Casal M. The use of genetically modified *Saccharomyces cerevisiae* strains in the wine industry. Applied Microbiology and Biotechnology. 2005;68(3):292–304. 10.1007/s00253-005-1994-2 15856224

[12] Wedral D, Shewfelt R, Frank J. The challenge of *Brettanomyces* in wine. LWT—Food Science and Technology. 2010;43(10):1474–9. 10.1016/j.lwt.2010.06.010 .

[13] Domizio P, Romani C, Lencioni L, Comitini F, Gobbi M, Mannazzu I, et al Outlining a future for non-*Saccharomyces* yeasts: Selection of putative spoilage wine strains to be used in association with *Saccharomyces cerevisiae* for grape juice fermentation. International Journal of Food Microbiology. 2011;147(3):170–80. 10.1016/j.ijfoodmicro.2011.03.020 . 21531033

[14] Rojas V, Gil JV, Pinaga F, Manzanares P. Studies on acetate ester production by non-*Saccharomyces* wine yeasts. International Journal of Food Microbiology. 2001;70(3):283–9. 10.1016/s0168-1605(01)00552-9 . 11764193

[15] Clemente-Jimenez JM, Mingorance-Cazorla L, Martinez-Rodriguez S, Heras-Vazquez FJL, Rodriguez-Vico F. Influence of sequential yeast mixtures on wine fermentation. International Journal of Food Microbiology. 2005;98(3):301–8. 10.1016/j.ijfoodchem.2004.06.007 . 15698691

[16] Moreira N, Mendes F, de Pinho RG, Hogg T, Vasconcelos I. Heavy sulphur compounds, higher alcohols and esters production profile of *Hanseniaspora uvarum* and *Hanseniaspora guilliermondii* grown as pure and mixed cultures in grape must. International Journal of Food Microbiology. 2008;124(3):231–8. 10.1016/ijfoodmicro.2008.03.025 . 18457893

[17] Crafack M, Mikkelsen MB, Saerens S, Knudsen M, Blennow A, Lowor S, et al Influencing cocoa flavour using *Pichia kluyveri* and *Kluyveromyces marxianus* in a defined mixed starter culture for cocoa fermentation. International Journal of Food Microbiology. 2013;167(1):103–16. 10.1016/j.ijfoodmicro.2013.06.024 . 23866910

[18] Saerens S, Swiegers JH, inventors; Google Patents., assignee. Enhancement of beer flavor by a combination of Pichia yeast and different hop varieties patent WO2013030398 A1. 2013 March.

[19] Pacheco A, Leão C, Almeida J, Santos J, Sousa MJ, Chaves S. The emerging role of the yeast Torulaspora delbrueckii in bread and wine production: using genetic manipulation to study molecular basis of physiological responses: INTECH Open Access Publisher; 2012. Available from: http://www.intechopen.com/books/export/citation/EndNote/structure-and-function-of-food-engineering/the-emerging-role-of-the-yeast-torulaspora-delbrueckii-in-bread-and-wine-production-using-genetic-ma.

[20] Ohshima Y, Sugaura T, Horita M, Sasaki T. Industrial application of artificially induced diploid strains of *Torulaspora delbrueckii*. Applied and Environmental Microbiology. 1987;53(7):1512–4. . 16347381 10.1128/aem.53.7.1512-1514.1987 PMC203901

[21] Birch AN, Petersen MA, Arneborg N, Hansen ÅS. Influence of commercial baker's yeasts on bread aroma profiles. Food Research International. 2013;52(1):160–6. 10.1016/j.foodres.2013.03.011.

[22] Birch AN, Petersen MA, Hansen ÅS. The aroma profile of wheat bread crumb influenced by yeast concentration and fermentation temperature. LWT—Food Science and Technology. 2013;50(2):480–8. 10.1016/j.lwt.2012.08.019.

[23] Almeida MJ, Pais C. Leavening ability and freeze tolerance of yeasts isolated from traditional corn and rye bread doughs. Applied and Environmental Microbiology. 1996;62(12):4401–4. . 8953712 10.1128/aem.62.12.4401-4404.1996 PMC168267

[24] Alves-Araujo C, Almeida MJ, Sousa MJ, Leao C. Freeze tolerance of the yeast *Torulaspora delbrueckii*: cellular and biochemical basis. Fems Microbiology Letters. 2004;240(1):7–14. 10.1016/j.femsle.2004.09.008 . 15500973

[25] Spano G, Russo P, Lonvaud-Funel A, Lucas P, Alexandre H, Grandvalet C, et al Biogenic amines in fermented foods. European Journal of Clinical Nutrition. 2010;64:S95–S100. 10.1038/ejcn.2010.218 . 21045859

[26] Abelson JN, Simon MI, Guthrie C, Fink GR. Guide to Yeast Genetics and Molecular Biology: Elsevier Science; 2004. 933 p.

[27] Burke D, Dawson D, Stearns T. Methods in yeast genetics: a Cold Spring Harbor Laboratory course manual. 2nd ed. New York: CSHL Press; 2000.

[28] Joosten H, Northolt MD. Detection, growth, and amine-producing capacity of lactobacilli in cheese. Applied and Environmental Microbiology. 1989;55(9):2356–9. . 16348016 10.1128/aem.55.9.2356-2359.1989 PMC203080

[29] Nikolaou E, Soufleros EH, Bouloumpasi E, Tzanetakis N. Selection of indigenous *Saccharomyces cerevisiae* strains according to their oenological characteristics and vinification results. Food Microbiology. 2006;23(2):205–11. 10.1016/j.fm.2005.03.004 . 16943006

[30] AACC. Approved Methods of Analysis. 11th Ed. Method 10–10.03. Optimized Straight-Dough Bread-Making Method. St. Paul, MN, USA: American Association of Cereal Chemists 2000.

[31] Meilgaard MC, Civille GV, Carr BT. Sensory evaluation techniques 3th ed. Florida, USA: CRC press; 1999.

[32] Plessas S, Fisher A, Koureta K, Psarianos C, Nigam P, Koutinas AA. Application of *Kluyveromyces marxianus*, *Lactobacillus delbrueckii* ssp. *bulgaricus* and *L*. *helveticus* for sourdough bread making. Food Chemistry. 2008;106(3):985–90. 10.1016/j.foodchem.2007.07.012.

[33] Halang WA, Langlais R, Kugler E. Cubic spline interpolation for the calculation of retention indices in temperature-programmed gas-liquid chromatography. Analytical Chemistry. 1978;50(13):1829–32. 10.1021/ac50035a026

[34] Adams RP. Identification of essential oil compounds by gas chromatography and mass spectrometry Illinois, USA: Allured Publishing Corporation; 2009.

[35] Mondello L. Wiley FFNSC Library—Mass Spectra of Flavors and Fragrances of Natural and Synthetic Compounds Shimadzu Corporation; 2011.

[36] Standard Reference Database [Internet]. 2011 [cited September 11, 2014]. Available from: http://www.nist.gov/srd/onlinelist.cfm.

[37] Rezaei MN, Dornez E, Jacobs P, Parsi A, Verstrepen KJ, Courtin CM. Harvesting yeast (*Saccharomyces cerevisiae*) at different physiological phases significantly affects its functionality in bread dough fermentation. Food Microbiology. 2014;39:108–15. 10.1016/j.fm.2013.11.013 . 24387860

[38] Flavornet and human odor space [Internet]. 2004. Available from: http://www.flavornet.org/.

[39] Pozo-Bayón MA, Guichard E, Cayot N. Flavor Control in Baked Cereal Products. Food Reviews International. 2006;22(4):335–79. 10.1080/87559120600864829

[40] Frauendorfer F, Schieberle P. Identification of the key aroma compounds in cocoa powder based on molecular sensory correlations. Journal of agricultural and food chemistry. 2006;54(15):5521–9. Epub 2006/07/20. 10.1021/jf060728k . 16848541

[41] Lee S-J, Noble AC. Characterization of Odor-Active Compounds in Californian Chardonnay Wines Using GC-Olfactometry and GC-Mass Spectrometry. Journal of agricultural and food chemistry. 2003;51(27):8036–44. 10.1021/jf034747v 14690393

[42] Rychlik M, Grosch W. Identification and Quantification of Potent Odorants Formed by Toasting of Wheat Bread. LWT—Food Science and Technology. 1996;29(5–6):515–25. 10.1006/fstl.1996.0079.

[43] Yang DS, Lee KS, Jeong OY, Kim KJ, Kays SJ. Characterization of volatile aroma compounds in cooked black rice. Journal of agricultural and food chemistry. 2008;56(1):235–40. Epub 2007/12/18. 10.1021/jf072360c . 18081248

[44] Schieberle P, Grosch W. Potent odorants of rye bread crust-differences from the crumb and from wheat bread crust. Zeitschrift für Lebensmittel-Untersuchung und Forschung. 1994;198(4):292–6. 10.1007/BF01193177

[45] Birch AN, Petersen MA, Hansen ÅS. Aroma of Wheat Bread Crumb. Cereal Chemistry Journal. 2014;91(2):105–14. 10.1094/CCHEM-06-13-0121-RW

[46] Schieberle P, Grosch W. Potent odorants of the wheat bread crumb Differences to the crust and effect of a longer dough fermentation. Zeitschrift für Lebensmittel-Untersuchung und Forschung. 1991;192(2):130–5. 10.1007/BF01202626

[47] Poinot P, Grua-Priol J, Arvisenet G, Rannou C, Semenou M, Bail AL, et al Optimisation of HS-SPME to study representativeness of partially baked bread odorant extracts. Food Research International. 2007;40(9):1170–84. 10.1016/j.foodres.2007.06.011.

[48] Annan NT, Poll L, Sefa-Dedeh S, Plahar WA, Jakobsen M. Volatile compounds produced by *Lactobacillus fermentum*, *Saccharomyces cerevisiae* and *Candida krusei* in single starter culture fermentations of Ghanaian maize dough. Journal of Applied Microbiology. 2003;94(3):462–74. 10.1046/j.1365-2672.2003.01852.x 12588555

[49] Busko M, Jelen H, Goral T, Chmielewski J, Stuper K, Szwajkowska-Michalek L, et al Volatile metabolites in various cereal grains. Food additives & contaminants Part A, Chemistry, analysis, control, exposure & risk assessment. 2010;27(11):1574–81. Epub 2010/08/24. 10.1080/19440049.2010.506600 . 20730644

[50] Frasse P, Lambert S, Richard-Molard D, Chiron H. The Influence of Fermentation on Volatile Compounds in French Bread Dough. LWT—Food Science and Technology. 1993;26(2):126–32. 10.1006/fstl.1993.1027.

[51] Verstrepen KJ, Derdelinckx G, Dufour JP, Winderickx J, Thevelein JM, Pretorius IS, et al Flavor-active esters: adding fruitiness to beer. Journal of bioscience and bioengineering. 2003;96(2):110–8. Epub 2005/10/20. . 16233495

[52] Saerens SM, Delvaux FR, Verstrepen KJ, Thevelein JM. Production and biological function of volatile esters in *Saccharomyces cerevisiae*. Microbial biotechnology. 2010;3(2):165–77. Epub 2011/01/25. 10.1111/j.1751-7915.2009.00106.x ; PubMed Central PMCID: PMCPmc3836583. 21255318 PMC3836583

[53] Christiaens Joaquin F, Franco Luis M, Cools Tanne L, De Meester L, Michiels J, Wenseleers T, et al The Fungal Aroma Gene *ATF1* Promotes Dispersal of Yeast Cells through Insect Vectors. Cell Reports. 2014;9(2):425–32. 10.1016/j.celrep.2014.09.009 25310977

[54] Lilly M, Lambrechts MG, Pretorius IS. Effect of Increased Yeast Alcohol Acetyltransferase Activity on Flavor Profiles of Wine and Distillates. Applied and Environmental Microbiology. 2000;66(2):744–53. 10.1128/aem.66.2.744-753.2000 10653746 PMC91891

[55] Birch AN, van den Berg FWJ, Hansen ÅS. Expansion profiles of wheat doughs fermented by seven commercial baker's yeasts. Journal of Cereal Science. 2013;58(2):318–23. 10.1016/j.jcs.2013.05.009.

[56] Brinker EM, Schmidt K, inventors; Google Patents, assignee. Proofing tolerant yeast-leavened dough patent US20100143534 A1. 2007 October.

[57] Hernandez-Lopez MJ, Prieto JA, Randez-Gil F. Osmotolerance and leavening ability in sweet and frozen sweet dough. Comparative analysis between *Torulaspora delbrueckii* and *Saccharomyces cerevisiae* baker's yeast strains. Anton Leeuw Int J G. 2003;84(2):125–34. 10.1023/a:1025413520192 . 14533716

[58] Maillard L. Action of amino acids on sugars. Formation of melanoidins in a methodical way. Comptes Rendus. 1912;154:66.

[59] McKinnon CM, Gelinas P, Simard RE. Wine yeast preferment for enhancing bread aroma and flavor. Cereal Chemistry. 1996;73(1):45–50. .

[60] Rossouw D, Jolly N, Jacobson D, Bauer FF. The effect of scale on gene expression: commercial versus laboratory wine fermentations. Applied Microbiology and Biotechnology. 2012;93(3):1207–19. Epub 2011/09/21. 10.1007/s00253-011-3564-0 . 21931974

[61] Steensels J, Snoek T, Meersman E, Nicolino MP, Voordeckers K, Verstrepen KJ. Improving industrial yeast strains: exploiting natural and artificial diversity. FEMS Microbiology Reviews. 2014;38(5):947–95. 10.1111/1574-6976.12073 24724938 PMC4293462

