# Supplementary figures and images for: Non-Conventional Yeast Strains Increase the Aroma Complexity of Bread
Source: PLoS One. 2016 Oct 24;11(10):e0165126. doi: 10.1371/journal.pone.0165126 (PMC5077118; doi:10.1371/journal.pone.0165126)

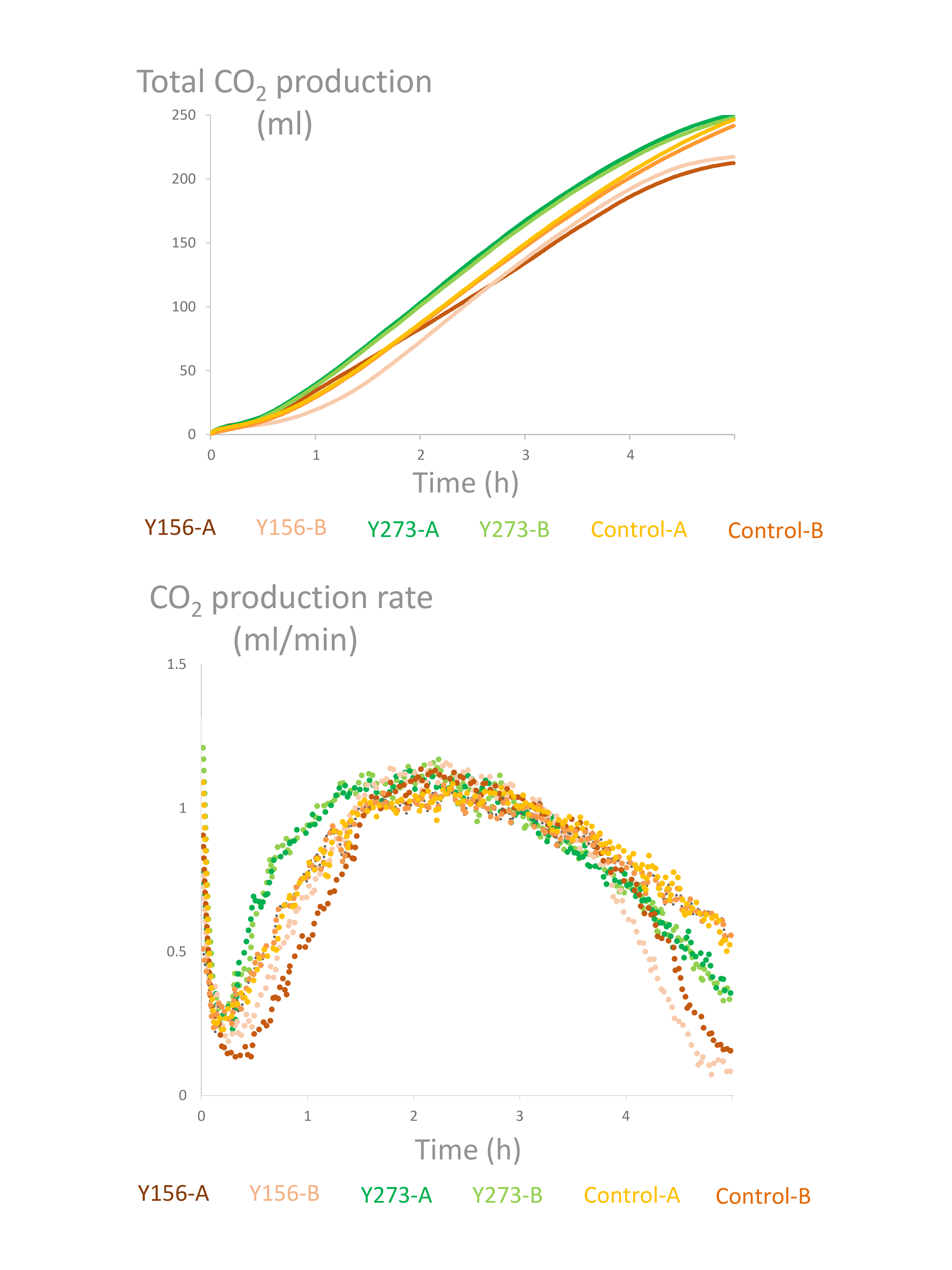

Supplement: S1 Fig — CO2 production of nonconventional yeasts Torulaspora delbrueckii (Y273) and S. bayanus (Y156) compared with a commercial bakery strain (control) as measured in the Risograph (replicates of each strain are identify as A and B). (TIF) [file pone.0165126.s001.tif]
